# Quantifying and Modeling the Impact of Phase State on the Ice Nucleation Abilities of 2-Methyltetrols as a Key Component of Secondary Organic Aerosol Derived from Isoprene Epoxydiols

**DOI:** 10.1021/acs.est.4c06285

**Published:** 2024-12-10

**Authors:** Xiaohan Li, Martin Wolf, Xiaoli Shen, Isabelle Steinke, Zhenli Lai, Sining Niu, Swarup China, Manish Shrivastava, Zhenfa Zhang, Avram Gold, Jason D. Surratt, Ian C. Bourg, Daniel J. Cziczo, Susannah M. Burrows, Yue Zhang

**Affiliations:** † Department of Civil and Environmental Engineering, Princeton University, Princeton, New Jersey 08544, United States; ‡ Yale School of the Environment, Yale University, New Haven, Connecticut 06511, United States; § Department of Earth, Atmospheric, and Planetary Sciences, Purdue University, West Lafayette, Indiana 47907, United States; ∥ Environmental and Molecular Sciences Laboratory, Pacific Northwest National Laboratory, Richland, Washington 99354, United States; ⊥ Department of Atmospheric Sciences, Texas A&M University, College Station, Texas 77843, United States; # Department of Environmental Sciences and Engineering, University of North Carolina at Chapel Hill, Chapel Hill, North Carolina 27599, United States; ¶ Department of Chemistry, University of North Carolina at Chapel Hill, Chapel Hill, North Carolina 27599, United States; ∇ High Meadows Environmental Institute, Princeton University, Princeton, New Jersey 08544, United States; + Atmospheric, Climate, and Earth Sciences Division, Pacific Northwest National Laboratory, Richland, Washington 99354, United States

**Keywords:** 2-methyltetrol, secondary organic aerosol, viscosity, ice-nucleating particles, heterogeneous nucleation

## Abstract

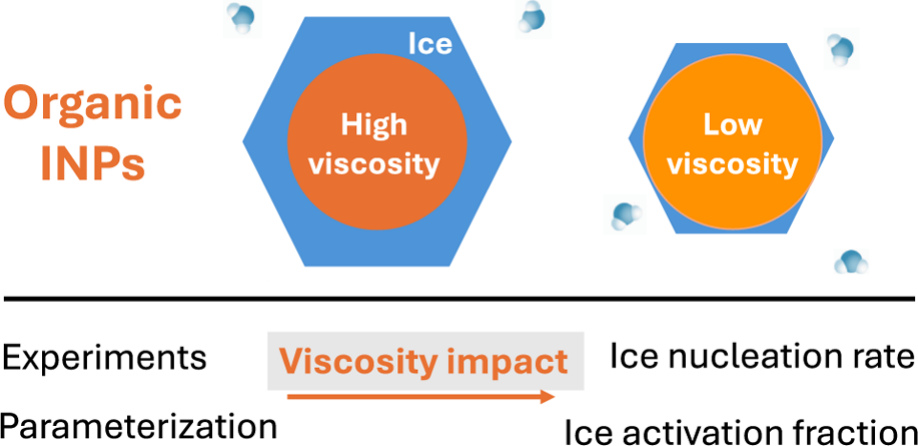

Organic aerosols (OAs) may serve as ice-nucleating particles (INPs), impacting the formation and properties of cirrus clouds when their phase state and viscosity are in the semisolid to glassy range. However, there is a lack of direct parameterization between aerosol viscosity and their ice nucleation capabilities. In this study, we experimentally measured the ice nucleation rate of 2-methyltetrols (2-MT) aerosols, a key component of isoprene-epoxydiol-derived secondary organic aerosols (IEPOX-SOA), at different viscosities. These results demonstrate that the phase state has a significant impact on the ice nucleation abilities of OA under typical cirrus cloud conditions, with the ice nucleation rate increasing by 2 to 3 orders of magnitude when the phase state changes from liquid to semisolid. An innovative parametric model based on classical nucleation theory was developed to directly quantify the impact of viscosity on the heterogeneous nucleation rate. This model accurately represents our laboratory measurement and can be implemented into climate models due to its simple, equation-based form. Based on data collected from the ACRIDICON-CHUVA field campaign, our model predicts that the INP concentration from IEPOX-SOA can reach the magnitude of 1 to tens per liter in the cirrus cloud region impacted by the Amazon rainforest, consistent with recent field observations and estimations. This novel parameterization framework can also be applied in regional and global climate models to further improve representations of cirrus cloud formation and associated climate impacts.

## Abstract 2

This study characterizes the impact of viscosity on the ice-nucleating ability of organic aerosols, with implications for cirrus cloud formation and climate predictions.

## 1 Introduction

Aerosols are liquid and solid particles suspended in the atmosphere.^1^ These particles, with sizes ranging from a few nanometers to tens of micrometers, have significant direct and indirect impacts on the Earth’s climate.^2−4^ Aerosol direct effects include their ability to scatter and absorb light. Aerosol indirect effects, also known as aerosol–cloud interactions, occur when aerosols serve as cloud condensation nuclei or ice nuclei, thereby altering cloud abundance and radiative properties.^2,3,5−7^ These indirect effects represent the largest source of uncertainty in estimating the climate impacts of aerosols.^2,3,8−10^

A key knowledge gap in aerosol–cloud interactions relates to how aerosols impact ice nucleation.^6,11−15^ Pure water vapor can nucleate ice at temperatures below about −38 °C in the natural environment.^16^ However, the presence of aerosols decreases the energy barrier for ice formation, leading to an increase in the onset temperature or reduction of the supersaturation with respect to ice (*S*_ice_) required for ice nucleation. Aerosol particles that can facilitate cloud ice formation under conditions where it would not normally occur are referred to as ice-nucleating particles (INPs).^17,18^ The presence of INPs, in turn, can significantly alter the optical and physical properties of clouds (e.g., cirrus clouds), leading to complex interplay between aerosols, clouds, and climate.^11,19−21^

In the atmosphere, mineral dust, organic particles, and inorganic salts have been shown to be effective INPs.^11,21−34^ Among them, mineral dust exhibits the widest range of conditions for the deposition ice nucleation (DIN) process, where water vapor directly forms ice on the surface of the INPs without the prior formation of liquid water, exhibiting onset temperatures between −10 and −60 °C with *S*_ice_ values ranging from 1 to 1.6.^11,22−26^ Inorganic salts, such as sea salt and ammonium sulfate, show ice nucleation capabilities similar to those of desert dust at cirrus cloud temperatures between −40 and −70 °C.^11,27−29^ Notably, organic aerosols (OAs) constitute the largest fraction of aerosol numbers in both the boundary layer and upper troposphere.^21,31−33^ Compared with other types of INPs, however, the impact of OAs on ice nucleation remains poorly understood due to their complex composition and variable phase state, combined with comparatively limited understanding of water–organic interactions.^5,21,35−42^

Organic aerosols account for up to 80% of submicron aerosol loading globally and are a significant component of the fine particulate matter (PM_2.5_).^43^ These particles exist in a range of phase states depending on temperature and relative humidity (RH), including liquid, semisolid, and solid (glassy) states.^44−50^ The phase state of OAs not only affects their chemical reactivity but also plays a pivotal role in their ice nucleation capability.^35,49,51^ Previously, OAs were often assumed to be in a liquid phase state, making them poor deposition INPs.^38^ However, recent studies from field, laboratory, and modeling perspectives have demonstrated that OAs can be glassy or semisolid under cirrus conditions and, thus, can significantly enhance heterogeneous ice nucleation.^20,35,40,45,52−57^ For example, previous studies have demonstrated that OAs in semisolid state can enhance ice nucleation and serve as efficient INPs.^20,40,55,56^ Some studies have also provided evidence that secondary organic aerosols (SOAs) in a glassy state serve as important sources of deposition INPs in the cirrus regime, especially when pre-exposed to low temperatures and humidity levels.^44,45,53,58,59^ Due to the high abundance of OA relative to other aerosols, even a small nucleation fraction can significantly enhance ice formation in the ambient environment.^13,21,60^ However, other studies have shown that SOA formed from selected precursors are ineffective INPs,^42,61^ possibly due to the varying phase states and morphologies of atmospheric OA particles arising from different chemical compositions, oxidation states, and environmental factors such as temperature and RH.^35,52^ The complicated relationship between the physicochemical properties and ice nucleation abilities of OA significantly hinders efforts to establish a general understanding of their role as INPs.^21,35,37,40,42,62^ In an effort to reduce this complexity, viscosity—an indicator of OA phase state—has routinely been used as a simple characteristic that may capture the ice nucleation properties of different organic species.^61,63^ However, previous theoretical attempts to represent the impact of phase state on the ice nucleation properties of OA have focused relatively narrowly on the kinetic impact of viscosity on water diffusion^35,64^ and have not yet resulted in a parametric model of heterogeneous ice nucleation rate suitable for use in regional and global atmospheric models.^13^

To bridge the aforementioned knowledge gap, this study combines experimental measurements with a semiempirical model to demonstrate the effect of phase state on OA’s ice nucleation abilities. We first present the experimental characterization of the ice nucleation abilities of OA as a function of the aerosol viscosity. For this study, we selected 2-methyltetrols (2-MT) as a representative component of OAs due to its abundance in tropospheric aerosols and potential role in DIN, particularly in the cirrus cloud region impacted by the Amazon forest. For instance, environmental chamber studies have demonstrated that 2-MT accounts for more than 33% of the total mass of SOA derived from isoprene-epoxydiol (IEPOX), highlighting its significance in ambient aerosols globally.^65−67^ Previous field measurements also confirm that SOA from IEPOX, including individual species, can serve as effective INPs.^20^ In regions including the Southeastern U.S. and the Amazon, IEPOX-derived SOA has been shown to contribute up to one-third of the total summertime OA.^33,68,69^ Herein, 2-MT was used as a sample compound to characterize the ice nucleation potential of OAs in this study. Then, we use classical nucleation theory together with a kinetic phase state model and experimental constraints to develop a semitheoretical representation of the effects of phase state on the ice nucleation rate of OAs. Finally, we apply our parameterization to estimate INP concentrations due to SOA over the Amazon and compare our predictions with field observations. This study presents a novel parameterization to systematically connect the physicochemical properties of OAs with their ice nucleation abilities, with potential applications in regional and global climate models to improve the understanding of the formation and properties of cirrus clouds and the accuracy of associated climate prediction.

## 2 Materials and Methods

### 2.1 Aerosol Generation and Phase State Control

**Figure 1:**
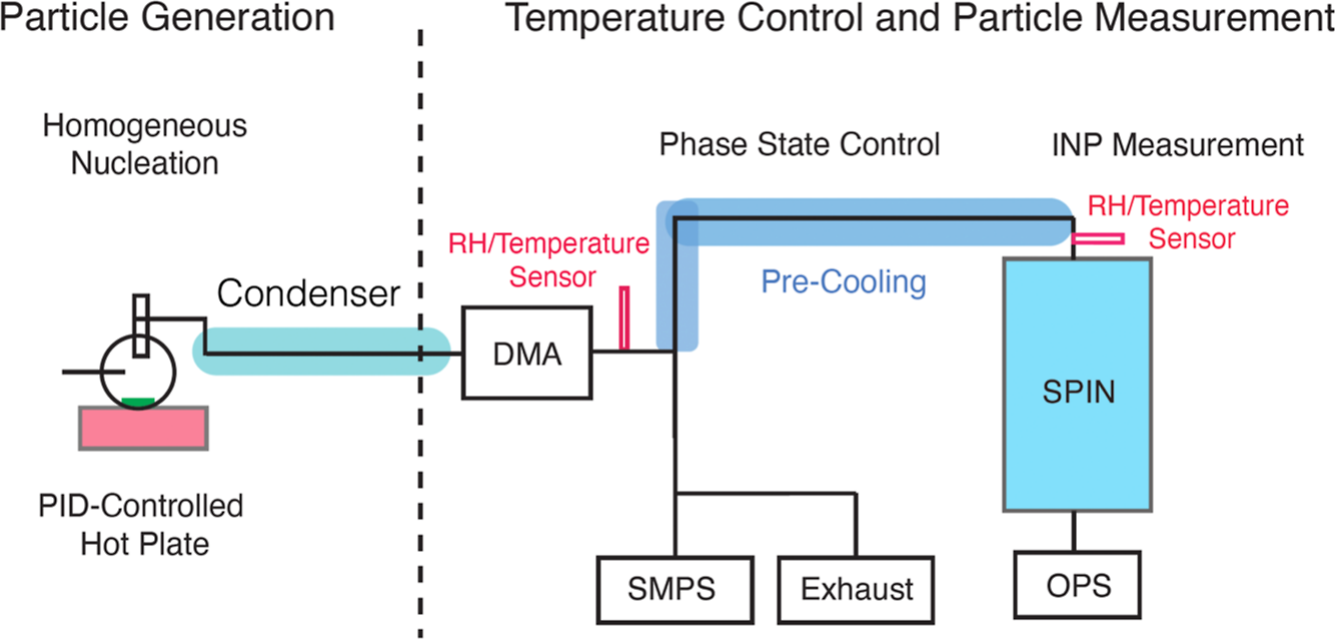
Schematic diagram of the experimental setup that includes OA generation, temperature and phase state control, and ice nucleation measurement.

OAs composed of 2-MT, a major component of isoprene-derived SOA,^70−75^ were generated using a temperature-controlled hot plate coupled with an evaporation and condensation apparatus. About 50 mg of 2-MT was synthesized in-house based on published procedures^76^ and placed by a micropipette at the bottom of the round flask. A proportional-integral-derivative (PID)-controlled heater (Omega, Inc.) was used to heat the round flask to 153 ± 0.1 °C to vaporize liquid 2-MT into gas phase. A gentle flow of zero air of 2 L/min passed through the round flask and carried the warm 2-MT vapor to a cooled condensing tube, where deionized water was used to cool the flow and induce self-nucleation of 2-MT aerosols, as shown in Figure 1.

The nucleated aerosols passed through a differential mobility analyzer (DMA; TSI Inc., model 3081) to select quasi-monodisperse particles of 100 nm mobility diameter with a sheath flow rate of 15 L/min and a sample flow rate of 1.5 L/min. These monodisperse particles were subsequently split into two lines with one line connected to a scanning mobility particle sizer (SMPS; TSI Inc., model 3080) and an exhaust with access flow. Another line of particles passed through a temperature-controlled region. The temperature in this region was controlled by a double-jacketed flow tube where the inner region allowed aerosols to pass through while the outer region was filled with recirculating ethanol. The temperature of the ethanol fluid was controlled between +20 and −55 °C by a circulation chiller (Lauda RP 3090 CW). The dew point of the air was measured to be −70 ± 1 °C by a Vaisala dew point sensor (DMT 152, Vaisala Inc.); therefore, the air did not reach supersaturation in the temperature-controlled region. A temperature sensor was installed at the end of the temperature-controlled region, right before the aerosol flow entered the SPectrometer for Ice Nucleation (SPIN, Droplet Measurement Technologies, Inc.) to precisely measure the aerosol flow temperature. The chiller temperature was adjusted to carry out experiments at aerosol flow temperatures of −25 °C, −5 °C, or 13 °C. The phase state of the aerosols was altered in this region by these different flow tube temperatures to generate either liquid or semisolid OAs for DIN measurements by the SPIN. We note that in the SPIN measurement, the temperature and RH experienced by the aerosol lamina can vary slightly due to minor heterogeneities in the SPIN conditions. These variations are monitored using 16 thermocouples along each SPIN wall. Since both conditions and uncertainties in temperature and RH are continuously fluctuating, the typical standard deviation of lamina temperature was less than 0.5 °C, which corresponds to an uncertainty in aerosol lamina RH of less than 4% of the RH with respect to ice (RH_ice_).

### 2.22.2 Deposition Ice Nucleation Measurement

The SPIN used for this study was a modified version of a commercially available continuous flow diffusion (CFDC) style instrument that has high sensitivity of activation fraction.^77,78^ Supersaturation *S*_ice_ and temperature within the nucleation section of the chamber were controlled by varying the temperature of two flat parallel walls separated by 1.0 cm and coated with approximately 0.1 cm of ice prior to each set of experiments. Temperature was monitored by using 16 thermocouples distributed along each wall. Slight variability in the temperature along each wall determined the uncertainty in the supersaturation achieved within the chamber.^79^ Aerosols were injected into the chamber using a knife-edge inlet, which nominally constrained the sampled aerosols to the centerline of the chamber. Particle-free sheath air was injected on both sides of the aerosol lamina at a 9:1 ratio of sheath to aerosol air flow rate.

In this study, *S*_ice_ was varied between 1.0 and 1.5, while temperature in the SPIN was controlled in the cirrus regime between −34 and −46 °C. For each precooling temperature of the aerosols, the experiments were conducted at four operation temperatures for the SPIN (i.e., −34, −38, −42, and −46 °C). At each SPIN operation temperature, *S*_ice_ was ramped from 1.0 to 1.5 and then down to 1.0 to symmetrically examine the DIN activity as a function of the aerosol phase state.

Turbulent mixing near the aerosol inlet caused some particles to spread outside of the aerosol lamina.^78^ Such spread exposed particles to a wider range of *S*_ice_ values than that of the lamina centerline, which translated to a lower activated fraction. To account for this phenomenon, the correction factors detailed in Wolf et al. (2018)^80^ were applied to the deposition nucleation data presented in this study.

Upon exiting the SPIN, particles entered the optical particle sizer (OPS) to record the side scattering and depolarization intensity for each particle within the size range of 0.5 to 15 μm. Only activated ice crystals and droplets were detected by the OPS as the size of the inactivated particles was below the cutoff limit for the OPS. A machine learning algorithm was used to differentiate activated liquid droplets and ice crystals based on scattering and depolarization signals of the particles detected by the OPS, as described in previous studies.^77,81^ Briefly, the SPIN OPS was equipped with a multivariate sensor that also detects three variables related to the depolarization of the incident laser light that was used to detect particle size.^77^ The detected particle size as well as these three variables were used by a machine learning algorithm to classify particles as either activated liquid droplets, activated INPs, or background frost shed from the iced walls. A SMPS was used to measure the total particle number concentration prior to activation, as shown in Figure 1. The activated fraction (*f*_ice_) was calculated as the number concentration of ice crystals derived from the OPS divided by the total particle number concentration measured by the SMPS at any given *S*_ice_. We use a number concentration of 1 L^–1^ as the lower bound of the OPS detection limit, resulting in the lower bound of the activation fraction of 10^–7^.^20^

We note that the onset RH in this study is defined as the RH at which ice nucleation becomes significant. It is an arbitrary value used in experiments to compare the ice nucleation properties of different aerosols. For mineral dust, the onset RH is often chosen when the frozen fraction reaches 1% or sometimes lower.^11^ In our study, we use 10^–5^ because SOA is typically much more abundant than mineral dust in number concentration, and thus, we do not require 1% of the SOA population to nucleate to generate a sufficient number of INPs in the atmosphere.

### 2.3 Phase State Characterization

The viscosity of OAs was quantified based on previous studies.^50,66,67,82^ Briefly, the temperature dependence of aerosol viscosity (η) is calculated by the Vogel–Tammann–Fulcher (VTF) equation^83,84^
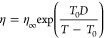
1where η_∞_ is the viscosity at infinite temperature, which takes the value of 10^–5^ Pa·s for modeling purposes;^84^*D* is the fragility parameter, which can be expressed empirically as a function of the molar mass of the organic molecules (*M*)^82^
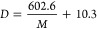
2and *T*_0_ is the Vogel temperature, which can be expressed as^84^
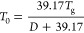
3where *T*_g_ is the glass transition temperature. The *T*_g_ values for OA at different RH values were estimated using the Gordon–Taylor equation^85^ as
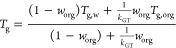
4where *T*_g,w_ is the glass transition temperature of pure water, which is suggested to be 136 K;^86^*k*_GT_ is the Gordon–Taylor constant for organic–water mixtures, which is suggested to be 2.5; *T*_g,org_ is the glass transition temperature of OAs under dry conditions, for which the previously measured value of 230 K for 2-MT was used in this study;^44,52,87,88^ and *w*_org_ is the mass fraction of organic matter (vs water) in the OA particles. This last parameter was evaluated using the relation , where  is the mass ratio of organic and water compounds, κ = 0.12 is the hygroscopicity of IEPOX-SOA,^66,89^ and *a*_w_ = RH/100 is the water activity. Finally, the relationship between RH and *S*_ice_ was calculated from the expressions for liquid water and ice saturation vapor pressure by Murphy and Koop.^90^

As illustrated in Figure 1, before entering the SPIN, dry 2-MT particles were precooled to specific temperatures to achieve desired phase state and viscosities in the semisolid-state range (10^2^ to 10^12^ Pa·s). Specifically, we derive the initial viscosities to be 8.2 × 10^7^ Pa·s, 1.6 × 10^5^ Pa·s, and 3.8 × 10^3^ Pa·s for 2-MT aerosol precooled to −25, −5, and 13 °C, respectively. During measurements, particle viscosity may decrease due to water diffusion into the particles. To account for this, we roughly estimated the water mixing time scale τ_mixing_^48^ as
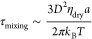
5where *D* = 100 nm is the diameter of 2-MT particles, η_dry_ is the viscosity of dry 2-MT particles, *T* is the SPIN operating temperature in Kelvin, and *a* is the hydrodynamic radius of the diffusing molecules. For water molecules, *a* = 0.144 nm,^91^ and the τ_mixing_ values are 1.6 × 10^4^ s, 30 s, and 0.67 s, respectively. When τ_mixing_ exceeds the particle residence time of 10 s in our experiment, as is the case for the semisolid particles at −25 °C and −5 °C, it is reasonable to assume that the particles maintained their phase state viscosity in the SPIN. For the case at 13 °C, where τ_mixing_ is less than 10 s, we adjusted the viscosity to reflect the wet state in equilibrium with the RH conditions within the SPIN. In other words, we anticipate that the particles adopted a liquid state with viscosities below 100 Pa·s.

We acknowledge that water diffusion can influence particle viscosity during ice nucleation, and in some cases, water diffusion into viscous OAs may occur much faster than predicted by the Stokes–Einstein equation under ambient conditions.^92,93^ However, it is important to consider the competing processes that occur simultaneously during ice nucleation: (1) the deposition of water on the particle surface as ice and (2) the diffusion of water into the particle. These processes interact with each other, and ice nucleation may occur before the particle reaches equilibrium with water if the nucleation occurs faster than the time scale of water diffusion. Future studies are needed to systematically examine the interplay between water diffusion and ice nucleation in OAs. Given the above-mentioned uncertainties, our evaluation of aerosol viscosity during the measurement provides a reasonable estimate, as ice nucleation can occur faster than the diffusion time scale. In the case which we treat the particle as wet, these particles are shown to have a mixing time scale an order of magnitude smaller than the residence time.

### 2.4 Relationship between Ice Fraction *f*_ice_ and Heterogeneous Ice Nucleation Rate *J*_het_

The heterogeneous ice nucleation rate, denoted *J*_het_, was calculated from the measured ice fraction *f*_ice_ according to the following derivation. In the case of isothermal droplet freezing experiments, the change in the number of unfrozen INPs, δ*N*_ufz_, during a time interval δ*t* can be expressed as
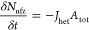
6Here, *J*_het_ represents the heterogeneous nucleation rate, with units of particles per surface area per time, and *A*_tot_ is the total surface area of all unfrozen INPs. For monodispersed particles, the total surface area can be expressed as
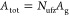
7where *A*_g_ denotes the surface area of a single INP.

From the combination of eqs 6 and 7, the relationship between the ice fraction *f*_ice_ and the nucleation time *t* of INPs can be formulated as
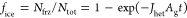
8In this equation, *N*_frz_ represents the number of frozen INPs and *N*_tot_ = *N*_ufz_ + *N*_frz_ is the total number of INPs. Our experimental aerosol residence time τ = 10 s and the aerosol particle size *d* = 100 nm are used to convert measured *f*_ice_ values to inferred *J*_het_ values.

### 2.5 Parameterization of *J*_het_

Under the framework of classical nucleation theory,^94^ the heterogeneous ice nucleation rate *J*_het_ can be expressed as
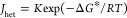
9where the exponential term accounts for thermodynamics of critical ice nucleus formation (ΔG* is the free energy change during the critical nucleus formation; *R* is the ideal gas constant; and *T* is absolute temperature), and *K* accounts for all kinetic processes associated with the formation of the nucleus.

For the thermodynamic processes, following classical nucleation theory, ΔG* depends on temperature *T* and ice saturation ratio *S*_ice_ as^95,96^
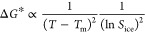
10where *T*_m_ is the melting temperature 273.15 K.

The kinetic term *K* is expected to scale with the number of water molecules *z* striking the surface of a suspended aerosol particle per unit time per surface area. From kinetic theory, this can be expressed as^1^
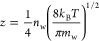
11where *n*_w_ is the number concentration of water molecules in the gas phase, *m*_w_ is the molecular weight of water, *k*_B_ is the Boltzmann constant, and *T* is the absolute temperature. From ideal gas law, *n*_w_ can be expressed as
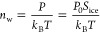
12where *P* is the water vapor pressure, *P*_0_ is the saturation water vapor pressure over ice in unit of Pa, and *S*_ice_ is the ice saturation ratio in the system. From the Clausius–Clapeyron equation, *P*_0_ can be expressed as^97^
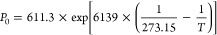
13

Based on eqs 9 through 13, we obtain the following prediction from classical nucleation theory and the kinetic theory of gases
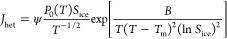
14where ψ and *B* are parameters to be determined. Attempts to fit our experimental results using eq 14 were unsuccessful because eq 14 is not sufficient to capture the widespread magnitudes of *J*_het_ induced by viscosity differences.

As noted above, several previous studies have hypothesized that the representation of aerosol ice nucleation ability can be improved by including a dependence on aerosol viscosity η. Here, we hypothesize that this can be represented by assuming that the kinetic variable *K*, in addition to its dependence on *z*, also depends on the aerosol water diffusion resistance. For simplicity, we model this dependence as a power-law relation
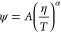
15where η is the viscosity of aerosol particles, and *A* and α are parameters to be determined. We note that the form of η/*T* is chosen based on the Stokes–Einstein–Sutherland equation, where the inverse of diffusion coefficient is 1/*D* ∝ η/*T*. Although eq 15 is empirical in its mathematical form, its use enables a preliminary evaluation of the manner in which diffusion within the particle may inhibit ice nucleation, e.g., through uptake of water into particles. When eq 14 is supplemented with eq 15, we obtain the following expression
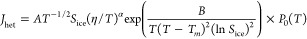
16where *J*_het_ has units of m^–2^ s^–1^, and *A*, *B*, and α are fitting parameters. The values of the parameters were optimized to obtain the best fit of high-quality experimental data with *S*_ice_ larger than a selected cutoff *S*_cut_ and *J*_het_ larger than the experimental detection uncertainty of ∼2 × 10^5^ m^2^ s^–1^ (see data uncertainty discussion in Section 3.2). We note that the fitted parameters are sensitive to the selection of *S*_cut_. Selecting *S*_cut_ within the range of 1.15 to 1.17 retains a sufficient amount of qualified data for statistical analysis and results in the most stable fitting parameters with *A* = 5.86 × 10^8^ (±1.00 × 10^8^), *B* = −7.7 × 10^4^ (∓ 0.4 × 10^4^), and α = 0.1359 (±0.0002). The fitted value of *B* yields a ratio of the heterogeneous and homogeneous nucleation free energy barriers, Δ*G*_hetero_*/Δ*G*_homo_* ≈ 0.24 at *S*_ice_ = 1.1, which is comparable to that of carbon surfaces and aligns well with the domain knowledge, indicating that organic carbon should enhance ice nucleation better than carbon surfaces with Δ*G*_hetero_*/Δ*G*_homo_* ≈ 0.45.^95^ The parameter α = 0.1359, as a positive value reflecting the impact of diffusion on ice nucleation rate, also matches well with our domain knowledge; that is, high viscosity and low diffusivity enhance heterogeneous ice nucleation even when OAs are in a semisolid or core–shell liquid state with viscosities below 10^12^ Pa s.^20,35,56^ Finally, the prefactor *A*, characterizing the magnitude of the *J*_het_ change with other variables, yields predictions that match experimental data well, as shown in Figure 3. Overall, the common domain knowledge indicates that the values determined here for *A*, *B* and *α* are reasonable and physically sound.

**Figure 2:**
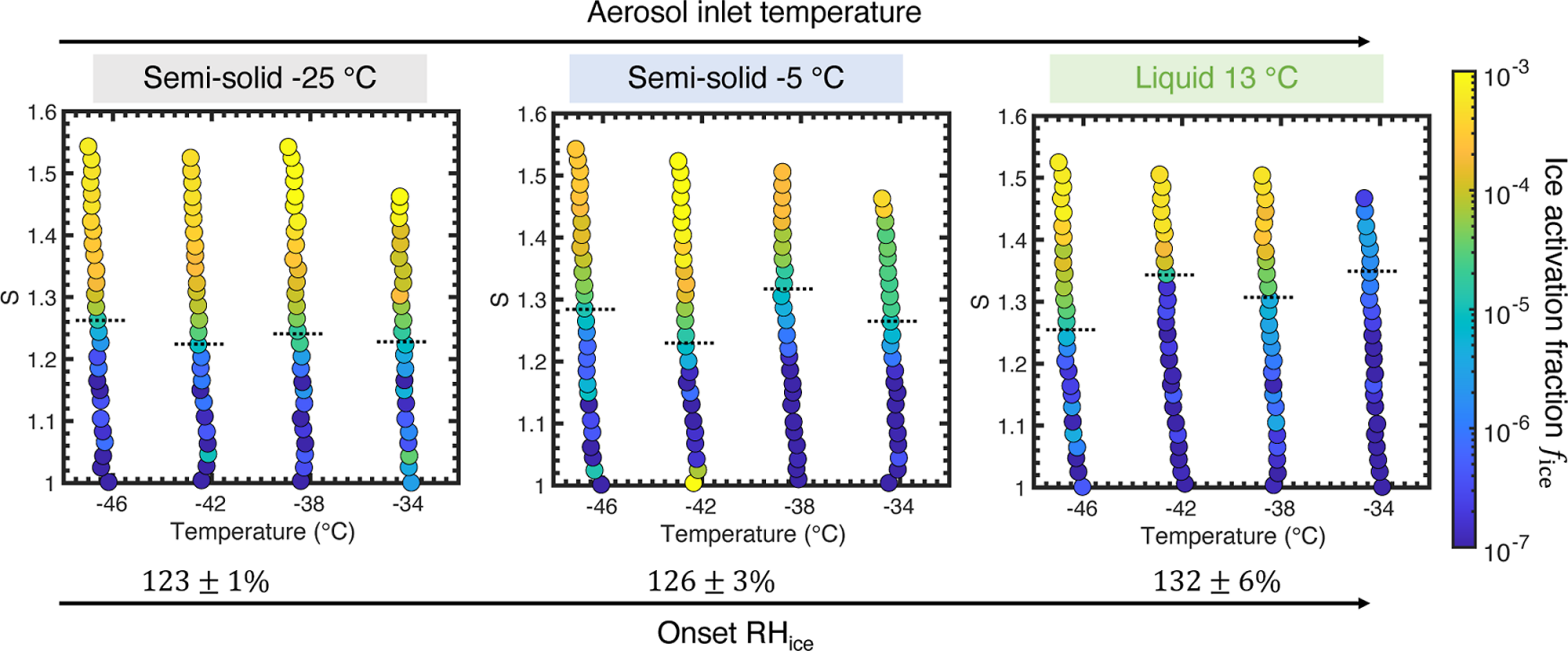
Ice activation fraction (*f*_ice_) for 2-MT particles plotted against experimental temperature and ice saturation ratio (*S*_ice_) in the SPIN. The three panels correspond to particles pre-equilibrated at different phase states through precooling temperatures of −25 °C (semisolid, η ≈ 8.2 × 10^7^ Pa·s), −5 °C (semisolid, η ≈ 1.6 × 10^5^ Pa·s), and 13 °C (liquid, η < 10^2^ Pa·s) as indicated by the top labels. Filled symbols represent experimental data points, with the colors indicating the measured ice activation fraction. Dashed black lines denote the onset of RH with respect to ice (RH_ice_), corresponding to *f*_ice_ = 10^–5^. The bottom labels show the average onset RH_ice_ for each precooling temperature.

**Figure 3:**
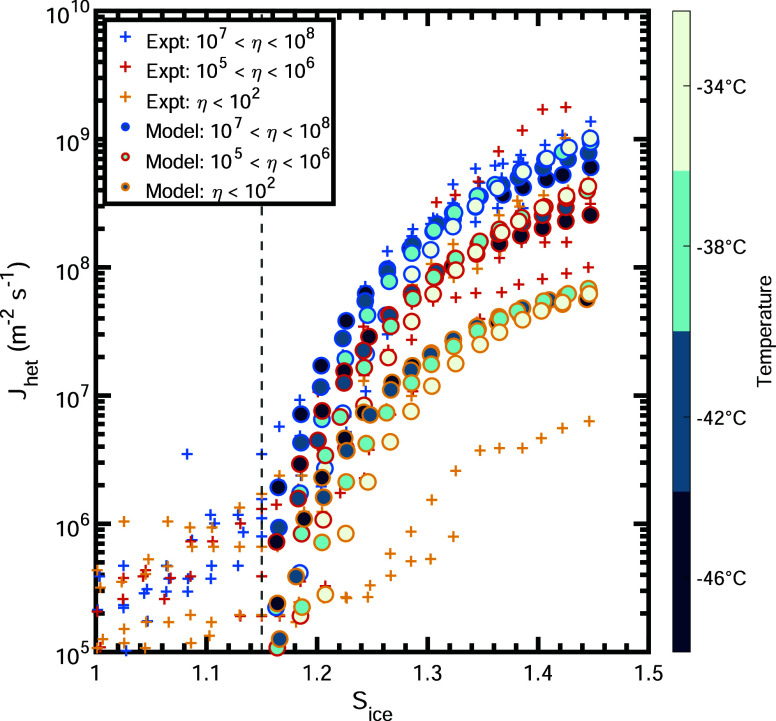
Heterogeneous ice nucleation rate (*J*_het_) as a function of ice saturation ratio (*S*_ice_) for 2-MT aerosol particles varying in phase state and experimental temperatures. Plus symbols (Expt) show *J*_het_ values derived from experimental ice fraction (*f*_ice_) as detailed in the methodology. Filled color symbols (Model) denote *J*_het_ as predicted by our parameterization; the fill color indicates the temperature, and the edge color corresponds to particle viscosity (i.e., phase state). The black dashed line indicates *S*_ice_ = 1.15.

We note that due to the logarithmic-scale nature of measured *J*_het_ values, as shown in Figure 3, the above-listed parameters were obtained by minimizing the sum of the squares of the residuals of the logarithm of the experimental ice nucleation rate *J*_het,expt_ and the predicted ice nucleation rate *J*_het,pred_ (see Supporting Information Section S1 for the discussion of using the L2 norm instead of the L1 norm for our parameterization). If the minimization is based, instead, on the sum of the squares of the residuals between *J*_het,expt_ and *J*_het,pred_, the fitting procedure (biased toward larger values of *J*_het_) yields *A* = 1.15 × 10^10^, *B* = −1.5 × 10^5^, and α = 0.0286, which increases predicted *f*_ice_ values by a factor of ∼2 in typical cirrus cloud conditions and is used in Section 3.4 to obtain an upper bound on the predicted *f*_ice_ values in field conditions. We note that the semitheoretical functional form shown in eq 15 is derived for any OA INPs and is independent of factors such as experimental procedures, OA components, and experimental conditions. Hence, this function is proposed to apply to atmospheric OA in general, and it is worth noting that the input viscosity represents the real time viscosity of the particles when the ice nucleation starts. However, the values of the fitting parameters (i.e., *A*, *B*, and α) were determined by fitting experimental data obtained in this study and hence are only rigorously valid for 2-MT SOA INP particles and in the range of the experimental conditions (e.g., *S*_ice_ ≥ 1.15, *T* ≤ −32 °C). Furthermore, the functional form used for the viscosity dependence in eq 15 is hypothetical, although consistent with observations suggesting that *J*_het_ increases with η. Our finding that the logarithmic-scale fitting yields α ≈ 0.14, whereas the linear-scale fitting yields α ≈ 0.03, suggests that the viscosity dependence of *J*_het_ may decay more rapidly at high η values than can be captured by a power-law relation.

## 3 Results and Discussion

### 3.1 Ice Activation Fraction *f*_ice_ from the Experiment

Figure 2 presents measured *f*_ice_ values for 2-MT particles across various phase states, temperatures, and *S*_ice_ conditions from the experiments. The uncertainty of measured *f*_ice_ values is on the order of 10^–7^ due to the detection limit of the SPIN. Typically, an activation fraction of 10^–2^ is used to characterize the onset of DIN for aerosol particles such as mineral dust and biological particles.^98−100^ In ambient environments including the upper troposphere, OAs can be orders of magnitude more abundant than mineral dust or nonorganic INPs in number concentration;^101,102^ therefore, a much lower activation fraction than 10^–2^ (e.g., 10^–5^) can still generate sufficient INPs to be comparable to those generated from other sources. It is worth noting that for an experiment with the liquid state and measurement temperature at −34 °C (i.e., the fourth dotted line in the right panel), the actual *f*_ice_ values did not achieve 10^–5^. Consequently, we extrapolated the onset RH_ice_, where *f*_ice_ = 10^–5^, using the increasing trend of *f*_ice_ when *S*_ice_ is between 1.3 and 1.4. Experimental results showed a significant rise in *f*_ice_, by 4 orders of magnitude, as *S*_ice_ increased from 1.0 to 1.5. Notably, our findings indicate that higher viscosity aerosols are more effective as INPs, as lower viscosity requires a higher *S*_ice_ to achieve an *f*_ice_ greater than 1 × 10^–5^.

### 3.2 Analysis of the Heterogeneous Ice Nucleation Rate (*J*_het_) in Experiments and through Parameterization

The relationship between the *J*_het_ values of 2-MT particles and *S*_ice_ is presented in Figure 3. Experimental *J*_het_ values are derived from experimental *f*_ice_ data with residence time τ = 10 s and particle diameter of 100 nm, as described in the Materials and Methods section. To examine the influence of aerosol phase state on ice nucleation, we classified both experimental (crosses) and predicted (circles) *J*_het_ values according to the corresponding viscosity ranges: semisolid (10^7^ < η < 10^8^ Pa·s), less semisolid (10^5^ < η < 10^6^ Pa·s), and liquid (η < 10^2^ Pa·s). It is worth noting that although viscosities between 10^2^ Pa·s and 10^12^ Pa·s are all considered to be semisolid, the time scale of water diffusion and the heterogeneous ice nucleation properties can vary significantly within such a wide viscosity range. We calculated the uncertainties of experimental *J*_het_ values to be on the order of ±2.3 × 10^5^ m^2^ s^–1^, corresponding to the *f*_ice_ uncertainty of ±10^–7^. As shown in Figure 3, when *S*_ice_ is less than 1.15, the *J*_het_ data are almost “flattened” around the detection limit with log-scale mean at *J*_het_ = 3.5 × 10^5^ m^2^ s^–1^ and highly uncertain. Hence, these data are not used for our parameterization, as discussed in the Materials and Methods section. Besides, below *J*_het_ values of ∼10^5^ m^2^ s^–1^, the ice frozen fraction is so small that ambient OA will not contribute to INP effectively, despite their relative high number concentration.

Our experimental findings indicate distinct behaviors of *J*_het_ under varying conditions: for *S*_ice_ ≤ 1.15, our measurement shows ice nucleation rates near or below the detection limit of our experimental setup, i.e., *J*_het_ values are small with unclear dependence on *T* and η; for 1.15 < *S*_ice_ < 1.3, there is a distinctive increase in *J*_het_ with *S*, manifesting as a steep slope of log *J*_het_ versus *S*_ice_. Viscosity begins to play a clearly significant role in the DIN process (see Supporting Information Figure S2 for a detailed view of the *J*_het_ dependence on η). Finally, for *S*_ice_ > 1.3, *J*_het_ continues to increase with *S*_ice_ but at a slower rate, while the impact of viscosity on *J*_het_ remains strong. Our model accurately reflects the last two distinct regions in the experimental data outlined above (where the experimental results are clearly statistically significant) and the notable trend that *J*_het_ increases with *S*_ice_ and η and decreases with environmental *T* (see Supporting Information Section S3 for details on the sensitivity of *J*_het_ to *S*_ice_, η, and *T*). We note that our model captures the general trend but does not capture the *J*_het_ values as accurately for one specific case with η < 10^2^, *T* = −34 °C, as shown by the right-most dotted vertical line in Figure 2 and the yellow crossed outliers in Figure 3. This inconsistency is likely associated with the experimental uncertainties discussed above and/or with the ice nucleation mechanism changing from DIN to other types of nucleation (e.g., immersion freezing).

As illustrated in Supporting Information Section S3 and Figure S3, our parameterization indicates that the phase state change of aerosols from the liquid state (η < 100 Pa·s) to the semisolid state (η up to ∼10^12^ Pa·s) can increase *J*_het_ by more than 2 orders of magnitude at *S*_ice_ ∼ 1.3 and temperature ∼ −40 °C. Overall, the alignment between our experimental data and model predictions effectively demonstrates the performance of our parameterization approach. Such results further emphasize the influence of viscosity on the ice nucleation rate when *S*_ice_ > 1.2 and temperature < −20 °C for 2-MT aerosols, with conditions aligning with typical cirrus cloud environments.

### 3.3 Assessing Parameterization: Prediction vs Observation

**Figure 4:**
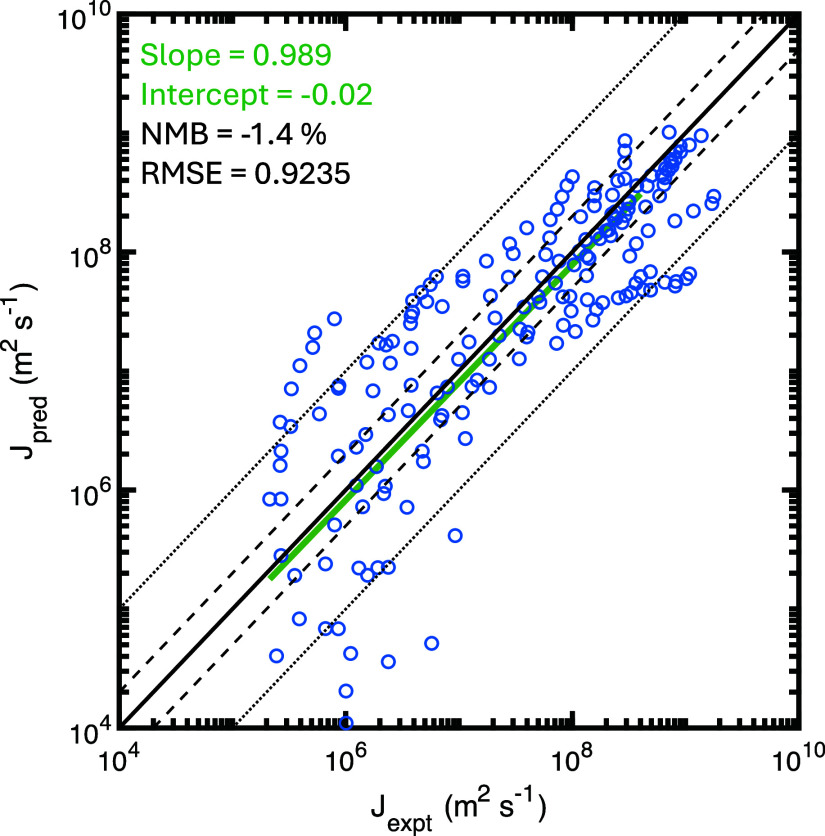
Comparison of predicted and experimental ice nucleation rate (*J*_pred_ vs *J*_expt_) in log–log scale. The blue points show the ice nucleation rate data. The black solid line indicates the location where *J*_pred_ = *J*_expt_. The black dashed and dotted lines represent differences by factors of 2 or 10, respectively. The green solid line shows the linear regression of blue data points for *J*_expt_ ranging from 1 × 10^5^ to 4 × 10^8^. The slope and intercept of this linear regression are displayed in green in the legend. The normalized mean bias (NMB) and root-mean-square error (RMSE) of log_10_*J*_pred_ vs log_10_*J*_expt_ are shown in black in the legend.

In Figure 4, we compare the predicted ice nucleation rate (*J*_pred_) from our parameterization with measurements (*J*_expt_) (see Supporting Information Figure S4 for the comparison of predicted vs measured *f*_ice_ values). Our model successfully captures a wide range of observed *J*_het_ (and *f*_ice_) values. Data points showing predicted vs experimental values are well centered around the 1:1 line (solid black line), indicating good agreement between predictions and observations. Our predictions show low values of normalized mean bias of NMB = −1.4% for log_10_*J* (and NMB = 1.6% for log_10_*f*). Furthermore, the linear regression of log_10_*J*_pred_ vs log_10_*J*_expt_ (and log_10_*f*_ice,pred_ vs log_10_*f*_ice,expt_) yields slopes of 0.989 (and 1.021), close to 1, demonstrating satisfactory agreement between our parameterization and the experimental data. We note that the *J*_pred_/*J*_expt_ values show a larger spread at lower *J*_expt_ values, which is likely due to the increased relative uncertainty in the experimental measurements at these lower ice nucleation rates. Overall, predicted values capture observations within 1 order of magnitude, when the *J*_het_ and *f*_ice_ values vary across more than 5 orders of magnitude.

### 3.4 Atmospheric Implications and Model Prediction of Atmospheric INPs from Organic Aerosols

**Figure 5:**
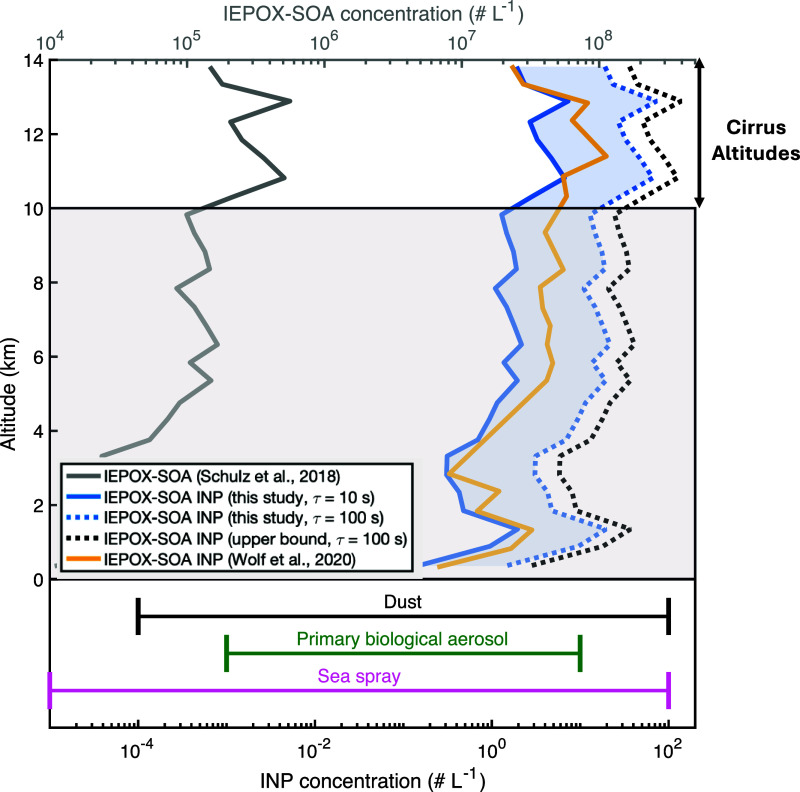
Vertical profiles of IEPOX-SOA and potential IEPOX-SOA INP number concentrations (# L^–1^) from 0 to 14 km altitude. The ranges for typical reported effective INP concentrations based on field measurements at various sites for different sources are shown below the 0 km altitude line: dust (black bar, 10^–4^ to 10^2^ L^–1^),^103−105^ primary biological aerosol (blue bar, 10^–3^ to 10^1^ L^–1^),^106−108^ and sea spray (pink bar, 10^–5^ to 10^2^ L^–1^).^109−111^ The gray solid line represents the IEPOX-SOA concentration derived from the ACRIDICON-CHUVA campaign over the Amazon in a tropical convective outflow system.^112^ The blue solid and dashed lines show potential IEPOX-SOA INP concentrations predicted by the parameterization (*A* = 5.86 × 10^8^, *B* = −7.7 × 10^4^, and α = 0.1359) for semisolid aerosol particles in equilibrium with the ambient environment with a viscosity of 1.47 × 10^3^ Pa·s at *T* = −46 °C and *S*_ice_ = 1.3 with residence time τ = 10 and 100 s, respectively; the black dashed line shows the upper bound limit of the potential IEPOX-SOA INP concentration developed in this study, with *A* = 1.15 × 10^10^, *B* = −1.47 × 10^5^, and α = 0.0286 and τ = 100 s at *T* = −46 °C and *S*_ice_ = 1.3; the yellow solid line corresponds to the prediction from Wolf et al.^20^ using *T* = −46 °C and *S*_ice_ = 1.3. The white background represents typical atmospheric altitudes where cirrus clouds form, while the shaded background below represents theoretically calculated INPs from IEPOX-SOA assuming cirrus conditions.

In the upper troposphere impacted by the Amazon rainforest, it is predicted (but not yet measured) that over 90% of IEPOX-derived SOA (IEPOX-SOA) mass is 2-MT,^113^ indicating the phase state of 2-MT could play a significant role in governing the DIN abilities of the aerosols in this region. Due to the likely high mass loading of 2-MT in these upper tropospheric aerosols, it is reasonable to use our parameterization for 2-MT to estimate the potential IEPOX-SOA INP concentration in the cirrus cloud regime of this region. To achieve this purpose, we use the vertical profiles of IEPOX-SOA concentration derived from the ACRIDICON-CHUVA (Amazon) field campaign (gray line in Figure 5), conducted in Manaus, Brazil, during September and October 2014.^112^

The yellow solid line in Figure 5 shows the estimated concentration of IEPOX-SOA INP by Wolf et al.^20^ at the condition *T* = −46 °C and *S*_ice_ = 1.3 based on the ambient IEPOX-SOA concentration (gray line). For comparison, we use the same IEPOX-SOA concentration and the same condition *T* = −46 °C and *S*_ice_ = 1.3 as Wolf et al. and plot our predictions at different aerosol residence times (τ) in Figure 5 (typical atmospheric τ values range from 10 to 100 s), assuming the particles are in equilibrium with the ambient environment. Our parameterization suggests that IEPOX-SOA INP concentrations at cirrus altitudes vary with aerosol residence time, ranging from approximately 1 to 6 L^–1^ when the residence time is 10 s (solid blue line) and from approximately 10 to 60 L^–1^ when the residence time is 100 s (dashed blue line) at −46 °C and *S*_ice_ = 1.3. Our predictions are comparable with the predictions by Wolf et al. (yellow solid line),^20^ which showed that the IEPOX-SOA INP concentration can reach 20 L^–1^ in the cirrus altitudes, despite the vast difference in methodology as our parameterization is based on laboratory and modeling results, while Wolf’s estimation was based on experimentally derived ice frozen fraction and the measurement of ambient IEPOX-SOA concentration. Moreover, the predicted potential INP concentrations from our parameterization are found to be comparable with prior measurements of ambient depositional INP concentrations ranging from 0.1 to 10^3^ L^–1^.^61,114,115^ We note that the predictions mentioned above are based solely on the parameterization of 2-MT from this study, and we did not include the more viscous 2-MT sulfates in our experiment. As our analysis is limited to 2-MT, the IEPOX-SOA in the natural environment—including 2-MT sulfates—might exhibit even greater ice nucleation activity. Hence, our results emphasize the potential significance of IEPOX-SOA (e.g., 2-MT) as a source of INPs in the upper troposphere, where IEPOX-SOA INP concentrations can potentially reach magnitudes of tens per liter. We note that the aging processes of SOAs, such as photoaging during their ascent through the atmosphere to cirrus regimes,^116^ can also impact the phase state and, consequently, the ice-nucleating abilities of SOAs.

As illustrated in Supporting Information Section S4 and Figure S5, we also performed another prediction of potential IEPOX-SOA INP concentration based on the ambient-measured meteorological data including temperature and RH from the ACRIDICON-CHUVA campaign. Our prediction shows that potential IEPOX-SOA INP particles are in the range of 1 to 46 L^–1^ at the altitude ∼13 km, which demonstrates IEPOX-SOA as a potential significant source of ambient INPs and aligns well with the above discussion. We note that in the atmosphere, the viscosity of INPs might change during the ice nucleation process. Hence, it is crucial to carefully characterize the viscosity before inputting these variables into our model. Proper characterization of the viscosity ensures that the model predictions accurately reflect the conditions, whether in the laboratory or the atmosphere.

Overall, our work provides a parameterization connecting viscosity with a heterogeneous ice nucleation rate from classic nucleation theory. Our study highlights the complex interplay between heterogeneous ice nucleation and the viscosity of 2-MT, and future studies are needed to further examine the viscosity dependence of heterogeneous ice nucleation of other types of OAs. This parameterization can be incorporated into regional and global-scale cloud-microphysical and climate models to systematically evaluate the impacts of SOA on ice nucleation. Future studies that incorporate such parameterizations to examine the time-dependent nucleation rates, kinetic effects of water diffusion during the ice nucleation processes, INP abilities of other SOA species, and INP concentrations from SOA of other regions such as the Southeastern U.S. are needed to further improve the understanding of cirrus cloud formation and associated climate impacts.

## Supporting Information Available

Section S1: discussion on choosing the L2 norm over the L1 norm for our parameterization; Section S2: viscosity dependence of *J*_het_ measured from experiments and predicted by our parameterization; Section S3: sensitivity of predicted *J*_het_ to ice saturation ratio, viscosity, and temperature; Section S4: model prediction of potential IEPOX INP concentration in meteorological conditions measured from the ACRIDICON-CHUVA campaign; Figure S1: histogram of predicted errors for *J*_het_ compared with the experiment; Figure S2: measured and predicted *J*_het_ as a function of *S*_ice_ at different η; Figure S3: predicted *J*_het_ as a function of *S*_ice_, η, and *T*; Figure S4: comparison of predicted and experimental ice nucleation rate (*f*_pred_ vs *f*_expt_) in log–log scale; Figure S5: meteorological condition and predicted potential IEPOX INP concentration for ACRIDICON-CHUVA campaign (PDF)
