# Supplementary material for: Quantifying and Modeling the Impact of Phase State on the Ice Nucleation Abilities of 2-Methyltetrols as a Key Component of Secondary Organic Aerosol Derived from Isoprene Epoxydiols
Source: Environ Sci Technol. 2024 Dec 10;58(51):22678–90. doi: 10.1021/acs.est.4c06285 (PMC11673119; doi:10.1021/acs.est.4c06285)
Supplement: Supplementary file 1 — es4c06285_si_001.pdf [file es4c06285_si_001.pdf]

Supporting Information submitted to Environmental Science and Technology

Quantifying and Modeling the Impact of Phase State on the Ice Nucleation Abilities of 2-Methyltetrols as a Key Component of Secondary Organic Aerosol Derived from Isoprene Epoxydiols

Xiaohan Li,[a,†*] Martin Wolf,[b] Xiaoli Shen,[c] Isabelle Steinke,[d,‡] Zhenli Lai,[e§] Sining Niu,[e] Manish Shrivastava[d], Swarup China,[f] Zhenfa Zhang,[g] Avram Gold,[g] Jason D. Surrat, [g,h] Ian C. Bourg[a,i], Daniel J. Cziczo,[c*] Susannah M. Burrows,[d*] Yue Zhang[e*]

[a] *Department of Civil and Environmental Engineering, Princeton University, Princeton, NJ, 08544*
[b] *Yale School of the Environment, Yale University, New Haven, CT, 06511*
[c] *Department of Earth, Atmospheric, and Planetary Sciences, Purdue University, West Lafayette, IN, 47907*
[d] *Atmospheric, Climate, and Earth Sciences Division, Pacific Northwest National Laboratory, Richland, WA, 99354, United States*
[e] *Department of Atmospheric Sciences, Texas A&M University, College Station, TX, 77843*
[f] *Environmental and Molecular Sciences Laboratory, Pacific Northwest National Laboratory, Richland, WA, 99354, United States*
[g] *Department of Environmental Sciences and Engineering, University of North Carolina at Chapel Hill, Chapel Hill, NC, 27599*
[h] *Department of Chemistry, University of North Carolina at Chapel Hill, Chapel Hill, NC, 27599*
[i] *High Meadows Environmental Institute, Princeton University, Princeton, NJ, 08544*
[†] *Currently at Atmospheric and Oceanic Sciences Program, Princeton University, Princeton, NJ, 08540*
[‡] *Currently at Department of Geoscience and Remote Sensing, Delft University of Technology, Delft, Netherlands, 2628 CN*
[§] *Currently at Environmental and Molecular Sciences Laboratory, Pacific Northwest National Laboratory, Richland, WA, 99354*

[*]Corresponding Authors: Yue Zhang, yuezhang@tamu.edu
Susannah M. Burrows, susannah.burrows@pnnl.gov
Daniel J. Cziczo, djcziczo@purdue.edu
Xiaohan Li, xiaohanl@princeton.edu

Summary: 7 pages, 4 sections, and 5 figures.

Contents:

Section S1: Discussion on choosing the L2 norm over the L1 norm for our parameterization.
Section S2: Viscosity dependence of J_{het} measured from experiments and predicted by our parameterization.
Section S3: Sensitivity of predicted J_{het} to ice saturation ratio, viscosity, and temperature.
Section S4: Model prediction of potential IEPOX INP concentration in meteorological conditions measured from the ACRIDICON-CHUVA campaign.
Figure S1. Histogram of predicted errors for J_{het} compared with experiment.

Figure S2. Measured and predicted heterogeneous ice nucleation rate (J_{het}) as a function of ice saturation ratio (S_{ice}) at different viscosities (η).

Figure S3. Predicted heterogeneous ice nucleation rate J_{het} as a function of (a) ice saturation ratio (S_{ice}), (b) viscosity (η), and (c) temperature (T).

Figure S4. Comparison of predicted and experimental ice nucleation rate (f_{pred} vs. f_{expt}) in log-log scale.

Figure S5. Vertical profiles of IEPOX-SOA number concentration, temperature, relative humidity (with respect to water), ice saturation ratio, and predicted potential IEPOX INP concentration for ACRIDICON-CHUVA campaign.

Section S1: Discussion on choosing the L2 norm over the L1 norm for our parameterization.

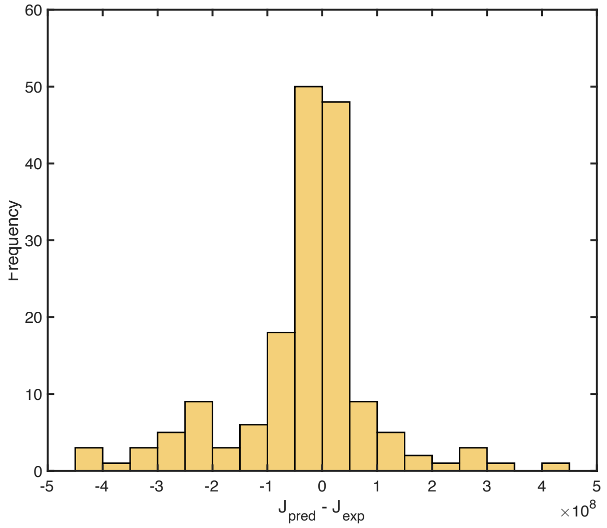

Figure S1. Histogram of predicted errors for J_{het} compared with experiment.

In our study, as discussed in Section 2.5 of the manuscript, we use the L2 norm to determine the fitting parameters by minimizing the sum of the squares of the residuals between predicted values and experimental values. This is in contrast to using the L1 norm, which involves minimizing the absolute difference between predicted values and experimental values. The choice between the L1 and L2 norms for determining parameters when fitting data to a functional form is a common question in statistical analysis and needs to be justified.

From a statistical perspective, whether a dataset can be better parameterized by the L1 or L2 norm is determined by theoretical analysis of the maximum likelihood estimation. The L1 loss is more suitable for datasets with noise exhibiting exponential decay, while the L2 loss is more suitable for datasets with Gaussian noise. Therefore, one way to determine which norm to choose is by plotting the histogram of prediction errors and observing how the errors are distributed. As shown in Figure S1, the error histogram of our parameterization is centered like a Gaussian distribution, which justifies our choice of the L2 norm.

Another justification for our choice comes from our procedure for performing parameterization. From Figure 3, we can see that almost all of the experimental outliers are in the range of $S = 1$ to $S = 1.1$. When we performed the parameterization, we used data with $S > 1.15$ to fit the parameters, where there are rare outliers. Hence, combining these two steps, the outliers

won't have much impact on our L2 norm parameterization.

Overall, our choice of the L2 norm in our parameterization is justified.

Section S2: The sensitivity of J_{het} to ice saturation ratio, viscosity, and temperature.

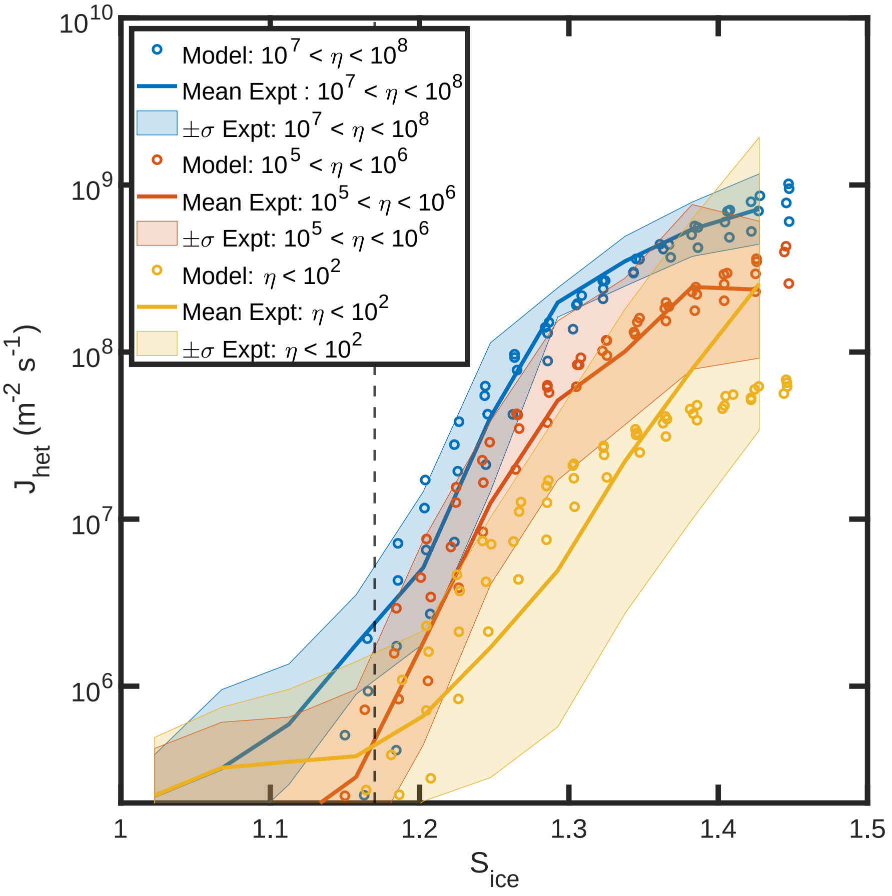

Figure S2. Measured and predicted heterogeneous ice nucleation rate (J_{het}) as a function of ice saturation ratio (S_{ice}) at different viscosities (η). The solid lines show the average experimental J_{het} values across four experimental temperatures at specific η ranges. The shaded areas show the $\pm\sigma$ spread for the average. The dotted symbols show the predicted J_{het} values at those four temperatures. The dashed black line shows the location of $S_{ice} = 1.17$.

As presented in Figure S2, the solid lines show the average values of experimental J_{het} at specific viscosity ranges. The shaded area shows the 1-sigma spread of the experimental data, and the circles show the predicted J_{het} values from the model. Although the experimental data have a large spread, the viscosity impact can be discerned from the averaged values of J_{het}, especially when $S_{ice} > 1.17$ (the range we focus on in the main manuscript). Additionally, this figure provides a validation of our predictions, as the model clearly captures the central behavior of the experimental viscosity dependence.

Section S3: The sensitivity of J_{het} to ice saturation ratio, viscosity, and temperature.

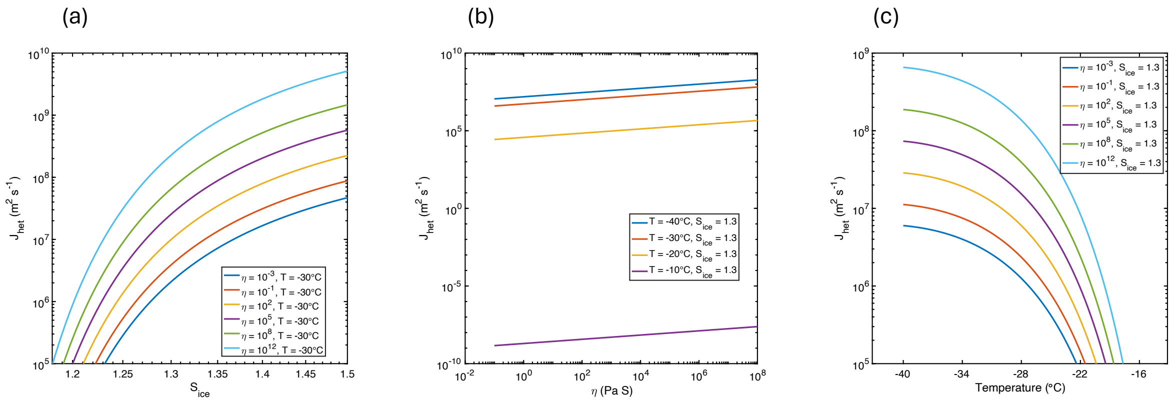

Figure S3. Predicted heterogeneous ice nucleation rate J_{het} as a function of (a) ice saturation ratio (S_{ice}), (b) viscosity (η), and (c) temperature (T).

Figure S3 illustrates the dependence of the J_{het} predicted by our parameterization on the ice supersaturation ratio (S_{ice}), viscosity (η), and temperature. From panels (a), (b) and (c), it is evident that J_{het} varies with viscosity (η), S_{ice} and temperature (T). At typical cirrus cloud conditions with $S_{ice} = 1.3$ and temperature around -40°C, the viscosity state of aerosols can increase the nucleation rate by two order of magnitude when comparing the liquid state ($\eta <$ 100 Pa s) to the semi-solid state (η up to 10^{12} Pa s). Overall, the impact of viscosity on ice nucleation is discernible and important when $S_{ice} > 1.2$ and temperature $<$ -20°C, which aligns with typical cirrus cloud conditions.

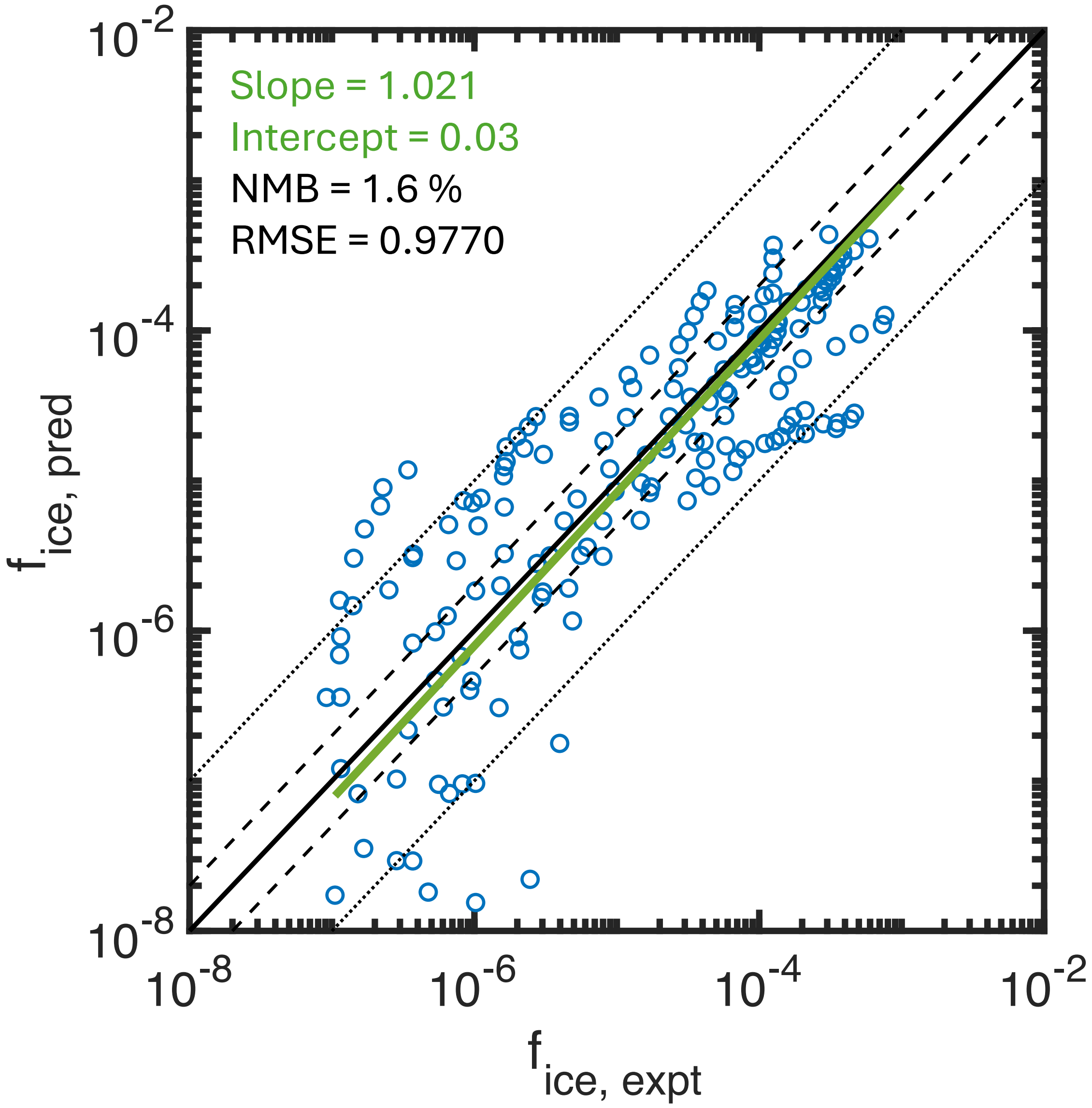

Figure S4. Comparison of predicted and experimental ice nucleation rate (f_{pred} vs. f_{expt}) in log-log scale. Blue circles represent the data points of predicted values versus experimental values. The black solid line indicates the location where $f_{pred} = f_{expt}$. The black dashed and dotted lines represent differences by a factors of 2 or 10, respectively. The green solid line shows the linear regression of blue data points for f_{expt} ranging from 1×10^{-7} to 1×10^{-4}. The slope and intercept of this linear regression are displayed in green in the legend. The normalized mean bias (NMB) and root mean square error (RMSE) of $\log_{10} f_{pred}$ vs. $\log_{10} f_{expt}$ are shown in black in the legend.

Section S4: Vertical profiles of IEPOX-SOA number concentration, temperature, relative humidity (with respect to water), ice saturation ratio, and predicted potential IEPOX INP concentration for ACRIDICON-CHUVA campaign.

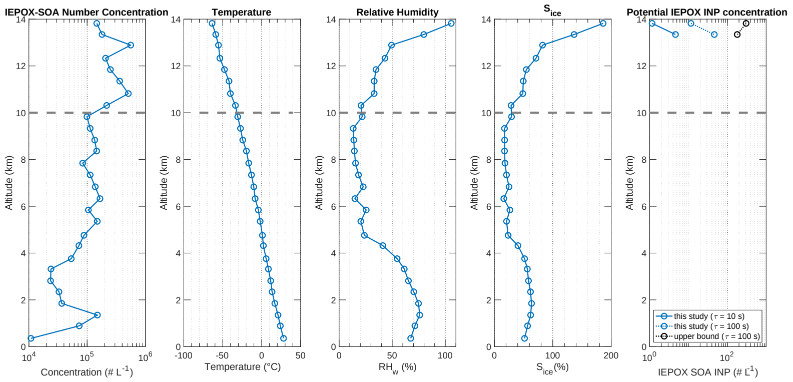

Figure S5. Vertical profiles of IEPOX-SOA number concentration, temperature, relative humidity (with respect to water), ice saturation ratio, and predicted potential IEPOX INP concentration for ACRIDICON-CHUVA campaign.

Figure S5 illustrates the vertical profiles of IEPOX-SOA number concentration, temperature, relative humidity with respect to water (RH_w) from the ACRIDICON-CHUVA campaign [1]. The corresponding ice saturation ratio and predicted potential IEPOX INP concentration are derived and illustrated in the right two panels. The blue line in the rightmost panel shows the prediction from our parameterization of Equation 15 in the manuscript with $A = 5.86 \times 10^8$, $B = -7.7 \times 10^4$, $\alpha = 0.1359$. The black line shows the upper bound prediction from our parameterization with $A = 1.15 \times 10^{10}$, $B = -1.47 \times 10^5$, $\alpha = 0.0286$ as discussed in Section 2.5. Again, our prediction under ambient meteorology conditions together with our predicted upper bound shows that the potential IEPOX SOA INP particles are in the range of 1 to 46 L^{-1} (with upper bound with τ = 100 s reaching 300 L^{-1}), which aligns well with the discussion in Section 3.4. We note that the predicted INP concentrations below 13 km are all 0 because the S_{ice} values are consistently less than 1 at altitudes below 13 km in this specific meteorological dataset. This falls outside the scope of our INP prediction parameterization, which is designed for conditions where $S_{ice} \geq 1$.

We emphasize that the theoretical functional form for organic aerosol (OA) INP nucleation rate (i.e., Equation 15) is valid for any OA INPs and is independent of experimental procedures, OA components, and experimental conditions, etc. However, we note that the values of the fitting parameters (i.e., A, B, α) are determined by fitting experimental data for 2-MT obtained in this study and hence would be only rigorously valid for 2-MT SOA INP particles and in the range of the experimental conditions (e.g., $S_{ice} \geq 1.15$, $T \leq -32$ °C).

References

1. Christiane Schulz, Johannes Schneider, Bruna Amorim Holanda, Oliver Appel, Anja Costa, Suzane S de Sá, Volker Dreiling, Daniel Fütterer, Tina Jurkat-Witschas, Thomas Klimach, et al. Aircraft-based observations of isoprene-epoxydiol-derived secondary organic aerosol (iepox-soa) in the tropical upper troposphere over the amazon region. *Atmospheric chemistry and physics*, 18(20):14979–15001, 2018.
